# Stereospecific Assay of (R)- and (S)-Goitrin in Commercial Formulation of Radix Isatidis by Reversed Phase High-Performance Liquid Chromatography

**DOI:** 10.1155/2017/2810565

**Published:** 2017-08-15

**Authors:** Lixing Nie, Zhong Dai, Shuangcheng Ma

**Affiliations:** National Institutes for Food and Drug Control, China Food and Drug Administration, 2 Tiantan Xili, Beijing, China

## Abstract

*Radix isatidis *(Banlangen) is a widely used traditional Chinese medicine for treating fever and removing toxic heat. Pharmacological studies have indicated that (R)-goitrin (epigoitrin) is one of the main constituents accounting for its antiviral activity, while (S)-goitrin (goitrin) is known as an antithyroid factor. To better control the quality of* radix isatidis *and its formulations, it is imperative to enantiomerically determine the contents of R- and S-goitrin. In this study, an enantioselective method based on reversed phase chromatography was developed for the assay of (R, S)-goitrin enantiomers. Optimum separation was obtained on an S-Chiral A column (4.6 mm × 250 mm, 5 *μ*m) using methanol/water (30 : 70, v/v) as the mobile phase. After validation, the method was applied to quantify the enantiomers in Banlangen granules, which is the most prescribed commercial formulation of* radix isatidis*. Compared to enantioselective resolution approaches based on normal phase chromatography, the new method could be conveniently performed using regular reversed phase high-performance liquid chromatography (RP-HPLC) equipment and was found to be more suitable for the enantioselective quality control of water-extracted and soluble medicines.

## 1. Introduction 

*Radix isatidis*, also known as Banlangen in Chinese, is a commonly used Chinese herbal medicine derived from the root of* Isatis indigotica Fort*. Pharmacological research has indicated that it has multiple biological activities [1–3], a major one being the antiviral effect [4–9]. In clinical practice,* radix isatidis* and its formulations have been employed for treating pestilence and seasonal toxin, fever, sore throat, macula, and papule caused by warm toxin, mumps, scarlatina, erysipelas facialis, erysipelas, and swelling abscess [10]. The most prescribed formulation of* radix isatidis* is Banlangen granules, which is regularly applied to prevent and treat a wide range of viral infections [11, 12].

**Figure 1:**
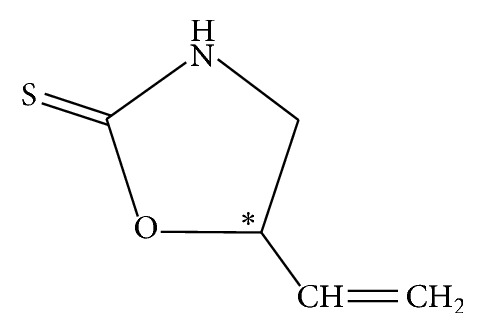
Chemical structures of (R, S)-goitrin.

Although the antiviral efficacy of Banlangen and its formulations is well acknowledged, the active ingredients have not been fully elucidated. To control the quality of related drugs, (R, S)-goitrin is assigned as the chemical marker for identification and assay in the Chinese Pharmacopoeia version 2015 [10]. Goitrin (5-vinyloxazolidine-2-thione) is a sulfur-containing alkaloid having a chiral carbon atom in position 2 of the oxazoline ring (Figure 1). It is commercially available as a mixture of (R)- and (S)-enantiomers. Results of chick embryo chorioallantoic membrane assay indicated that (R)-goitrin (epigoitrin) is the main active constituent in Banlangen [13], whose inhibitory effect against influenza viral neuraminidase was revealed by fluorescent enzyme immunoassay method [14]. However, (S)-goitrin (goitrin) is known as an antithyroid factor [15]. Since the pharmacological effect is mainly related to the (R)-enantiomer, there is a need to develop enantioselective methods to resolve (R)- and (S)-goitrin for the effective use and quality control of* radix isatidis* and its formulations.

High-performance liquid chromatography (HPLC) has been the most commonly used technology for the enantioseparation of drugs, due to its ease of use and the commercial availability of columns [16, 17]. Gas chromatography (GC) [18, 19], capillary electrophoresis (CE) [20, 21], and nuclear magnetic resonance spectroscopy (NMRS) [22, 23] also play important roles in the chiral recognition and analysis of enantiomers. Furthermore, the need for high-throughput and high-efficiency enantioseparation has led to the advancement of chiral screening techniques, such as supercritical fluid chromatography (SFC) [24, 25] and high-speed countercurrent chromatography (HSCCC) [26, 27]. However, there have been fewer reports on the stereospecific resolution and analysis of enantiomers in natural products [28].

In 2010, (R, S)-goitrin were first resolved by the current authors using normal phase liquid chromatography (NPLC) [29], and the method was applied to the chiral analysis of* radix isatidis*. Later, we developed an SFC methodology for the fast and high-throughput separation and preparation of (R)- and (S)-goitrin [30]. However, the above two protocols are based on the normal phase chromatographic system, which uses low-polarity solvents such as* n*-hexane and CO_2_ as the mobile phase. Therefore, they are not appropriate for analyzing water-soluble samples. Meanwhile, commercial formulations of* radix isatidis* are produced via water extraction and alcohol precipitation techniques. Samples of these formulations cannot be dissolved in organic reagents. If the samples are first dissolved with water, the test solutions may precipitate during elution when analyzed by NPLC or SFC methods. In this respect, it is necessary to establish an approach based on reversed phase chromatography (which uses an aqueous mobile phase) for the stereospecific assay of (R)- and (S)-goitrin in commercial formulations of* radix isatidis*.

In this study, a simple, convenient, and accurate reversed phase liquid chromatography (RPLC) method was developed to resolve (R, S)-goitrin. The separation conditions were optimized, and the method was validated and applied to quantify (R)- and (S)-goitrin in commercial Banlangen granules.

## 2. Materials and Methods

### 2.1. Chemicals and Reagents

Methanol and dimethylsulfoxide of HPLC grade were purchased from Merck (Darmstadt, Germany). (R, S)-Goitrin reference (product number 111753) was obtained from National Institutions for Food and Drug Control (Beijing, China) with content of 100%. (R)-Goitrin and (S)-goitrin were previously prepared by the SFC method [30]. All the experiments used purified water from a Direct-Q 3 system (Millipore, Bedford, USA). Banlangen granules from 9 different batches and two manufacturers were purchased from local drug stores (Beijing, China).

The stock and working solutions containing the racemic mixture of (R, S)-goitrin were prepared with methanol. Individual solutions of (R)- and (S)-goitrin for peak identification were prepared separately with dimethylsulfoxide.

### 2.2. Apparatus and Analytical Conditions

The analyses were performed on a Shimadzu LC2010AHT system (Kyoto, Japan) equipped with a pump, a system controller, and a UV-vis detector operating at 245 nm. The software LCsolution was used for data acquisition. An S-Chiral A column (4.6 mm × 250 mm, 5 *μ*m) obtained from Acchrom (Beijing, China) was employed for the enantiomer separation. The mobile phase was comprised of methanol and water. The injection volume was 10 *μ*L for all solutions. The portion of mobile phase, column temperature, and flow rate were optimized to obtain the best resolution. The migration order was established by analyzing the pure (R)- and (S)-goitrin enantiomers separately.

### 2.3. Preparation of Banlangen Granules for Assay

Granules (1 g) were weighed accurately and transferred into a 25 mL volumetric flask, into which approximately 20 mL of water was added. The mixture was then sonicated for 5 min and allowed to rest for 5 min before bringing it up to volume. The solution was filtered through a 0.45-*μ*m filter.

### 2.4. Method Validation

The method was validated for (R, S)-goitrin enantiomer analysis in standard solutions and granule samples as per the recommendations laid down by International Conference on Harmonization (ICH) guideline [31]. The linearity of the method was determined by the construction of calibration curves using five concentration levels. Three replicate injections of the standard solutions were made, and the peak area versus the concentrations of each enantiomer was plotted. The obtained data were then subjected to regression analysis by the least-squares method to calculate the calibration equation and correlation coefficient (*r*). The limit of detection (LOD) and limit of quantitation (LOQ) were separately determined at the signal-to-noise ratio (S/N) values of 3 and 10, respectively. For precision test, the intraday and interday variations were determined at three different concentration levels. Three replicates were performed for each concentration. To confirm the repeatability, six test solutions were prepared from the same sample and analyzed. The recovery test was performed by the standard addition method. 0.5 g of the sample and three different volumes of a standard solution of (R, S)-goitrin at low, middle, and high concentration levels were added to a 25-mL volumetric flask. The mixture was extracted and analyzed by the proposed procedure. Triplicate experiments were performed at each level.

## 3. Results and Discussion

### 3.1. Method Optimization

To obtain chromatograms with better resolution of enantiomers within a shorter time, the chromatographic conditions were optimized. The ratio of methanol in the mobile phase, the flow rate, and column temperature were chosen as optimization factors. The retention time (Rt) of (R)- and (S)-goitrin and the resolution (Rs) were used as criteria to evaluate the quality of separation.

**Figure 2:**
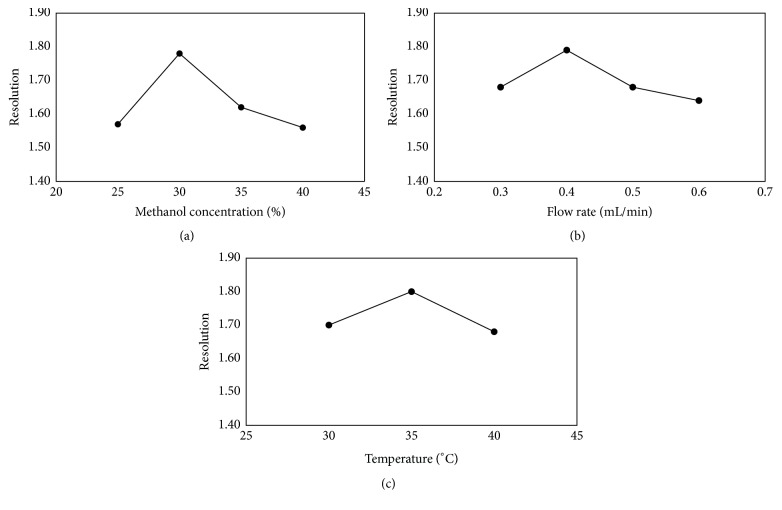
Effect of (a) methanol concentration, (b) flow rate, and (c) column temperature on the resolution of (R, S)-goitrin enantiomers. Experimental conditions: S-Chiral A column (4.6 mm × 250 mm, 5 *μ*m), methanol/water, 245 nm, and injection volume of 10 *μ*L.

**Figure 3:**
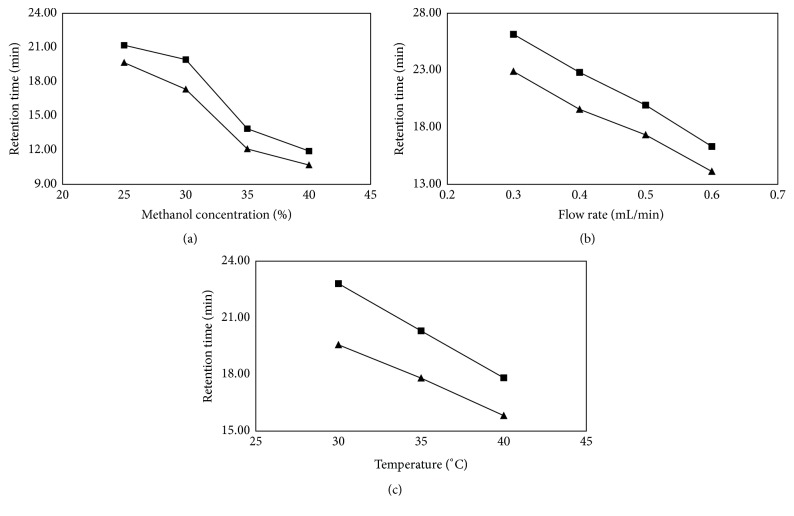
Effect of (a) methanol concentration, (b) flow rate, and (c) column temperature on the retention time of (R)-goitrin (▲) and (S)-goitrin (■). Experimental conditions: S-Chiral A column (4.6 mm × 250 mm, 5 *μ*m), methanol/water, 245 nm, and injection volume of 10 *μ*L.

Methanol concentration is crucial for achieving optimal separation. As shown in Figure 2(a), the resolution increased with decreasing methanol concentration to reach a maximum level, beyond which the resolution was reduced. Since the retention time (Figure 3(a)) also increased with decreasing methanol concentration, the concentration of methanol in mobile phase was chosen to be 30% (v/v).

The flow rate of the mobile phase is another important parameter to be controlled. According to Figure 2(b), a decrease in the flow rate increased the resolution, until an inflection point appeared. At the same time, there was sustained growth in the retention time (Figure 3(b)). The best resolution with a lower retention time was obtained at the flow rate of 0.4 mL/min.

In addition, the effect of column temperature in the range from 30 to 40°C was investigated. Increasing the column temperature caused a slight change in resolution and a significant decrease in retention time (Figures 2(c) and 3(c)). Based on the results, 35°C was selected as the optimal value for the other experiments.

After optimizing all three parameters, the best conditions for the enantioseparation of (R, S)-goitrin (in terms of successful elution and resolution) are presented in Table 1. The migration order of the enantiomers is the same as that from the reported SFC method but is the opposite to that from the NPLC method [29, 30].

### 3.2. Method Validation

Linear regression analyses were performed by plotting the ratio of peak area (*y*) versus the concentrations of the injected standard enantiomer (*x*). A linear relation was observed over the concentration range for each enantiomer, with correlation coefficient *r* > 0.995. The LOD (S/N ≥ 3) and LOQ (S/N ≥ 10) of (R)- and (S)-goitrin were down to ng/mL levels. The obtained intra- and interday precisions, repeatability, and accuracy are presented in Table 2. The overall relative standard deviation (RSD) was less than 3%; and the average recoveries for both enantiomers were close to 100%. Therefore, this RPLC method is precise, accurate, and sensitive for the quantitative determination of the studied drug.

### 3.3. Stereospecific Analysis of Commercial Formulation

**Figure 4:**
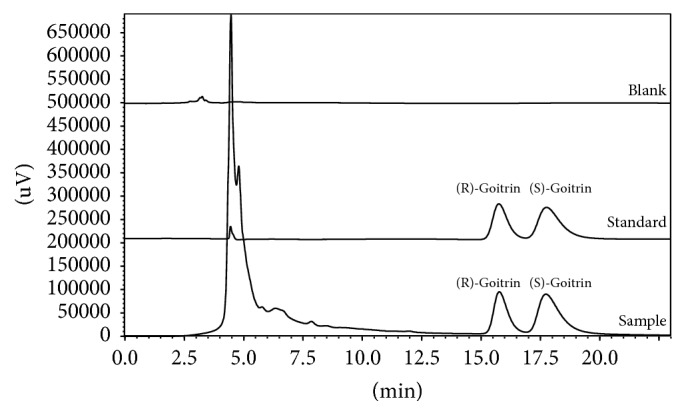
Typical chromatograms obtained for the blank reference, standard solution containing racemic (R, S)-goitrin, and commercial granules. Experimental conditions: S-Chiral A column (4.6 mm × 250 mm, 5 *μ*m), methanol/water (30 : 70, v/v), 0.4 mL/min, 35°C, 245 nm, and injection volume of 10 *μ*L.

The method developed above was applied to determine the (R, S)-goitrin enantiomers in commercial Banlangen granules. The chromatographic peaks were identified by comparing the retention times and UV spectra with those of (R)- and (S)-goitrin references. Spiking the samples with the reference compounds did not lead to any additional peaks, which further confirmed the identities of the peaks. The pharmaceutical formulation excipients in Banlangen granules did not interfere with the assay (Figure 4).

Nine batches of Banlangen granules from two manufacturers were tested. As shown in Table 3, the samples show remarkable differences in the contents of (R)- and (S)-goitrin. The content of (R)-goitrin had a wide range of 0.024–0.494 mg/g and the relative standard deviation (RSD) of the content in nine samples reached to 97.4%, while that of (S)-goitrin had a range of 0.018–0.429 mg/g with a high RSD of 106.8%. This considerable variation might arise from the variable quality of the* radix isatidis* raw material and unstable manufacturing technology. On the contrary, the ratio between the (R)- and (S)-isomers in each sample was more consistent, which could be attributed to the relatively stable natural distribution of the enantiomers in* radix isatidis*. In addition, the total amount of (R)- and (S)-goitrin determined by the enantioselective RP-HPLC method was close to the manufacturer data, that is, the content of (R, S)-goitrin in the same batches of samples determined by routine nonenantioselective HPLC method. And this in turn indicated that the newly proposed method was accurate.

## 4. Conclusion

In summary, a novel RP-HPLC method was developed for the separation of (R, S)-goitrin and used to quantify these enantiomers in commercial formulations of* radix isatidis*. Good performance was achieved in terms of resolution, linearity, LOD, LOQ, precision, and accuracy. In addition, the method could be conveniently performed on a regular reversed HPLC system for analyzing water-extracted samples. Great variations among samples from different factories and batches were found, which will be further investigated. The accurate measurement method reported here could allow better control of the internal quality of commercial formulations of* radix isatidis* and help identify possible reasons of the quality variation and design appropriate countermeasures.

## Figures and Tables

**Table 1:** Optimized conditions for (R, S)-goitrin enantiomer separation.

Parameter	Optimization result
Stationary phase	S-Chiral A column (4.6 mm × 250 mm, 5 *μ*m)
Column temperature	35°C
Mobile phase	Methanol/water (30 : 70, v/v)
Flow rate	0.4 mL/min
Detection wavelength	245 nm
Injection volume	10 *μ*L

**Table 2:** Linearity, LOD, LOQ, precision, repeatability, and recovery of the RPLC separation method.

	S-Goitrin			R-Goitrin		
Linearity range (*μ*g/mL)	0.08, 0.4, 2, 8, 40			0.1, 0.6, 3, 12, 60		

LOD (ng/mL)	2.0			2.9		
LOQ (ng/mL)	81.9			118.0		

Precision						
Concentration level (*μ*g/mL)	0.08	2	40	0.1	3	60
Intraday (*n* = 6, RSD, %)	0.6	0.3	0.7	0.6	0.8	1.1
Interday (*n* = 3, RSD, %)	0.9	0.5	1.0	1.3	0.5	0.4

Repeatability (*n* = 6, RSD, %)	2.1			2.6		

Recovery						
Mean (%)	100.1			99.4		
RSD (*n* = 9, %)	0.5			0.3		

**Table 3:** Contents of (R)- and (S)-goitrin in 9 batches of Banlangen granules (*n* = 3).

Sample	(R)-Goitrin (mg/g)	Standard deviation (mg/g)	(S)-Goitrin (mg/g)	Standard deviation (mg/g)	R/S ratio
A1	0.071	0.0009	0.037	0.0005	1.93
A2	0.247	0.0040	0.174	0.0026	1.42
A3	0.209	0.0019	0.183	0.0018	1.14
A4	0.494	0.0114	0.429	0.0090	1.15
A5	0.272	0.0044	0.199	0.0034	1.37
A6	0.079	0.0008	0.056	0.0005	1.41
B1	0.024	0.0002	0.018	0.0001	1.30
B2	0.026	0.0002	0.020	0.0002	1.33
B3	0.039	0.0004	0.030	0.0003	1.30

Mean	0.162		0.127		1.37
RSD (%)	97.4		106.8		17.0
